# Bovine spongiform encephalopathy infection alters endogenous retrovirus expression in distinct brain regions of cynomolgus macaques (*Macaca fascicularis*)

**DOI:** 10.1186/1750-1326-6-44

**Published:** 2011-06-23

**Authors:** Alex D Greenwood, Michelle Vincendeau, Ann-Christin Schmädicke, Judith Montag, Wolfgang Seifarth, Dirk Motzkus

**Affiliations:** 1 Leibniz-Institute for Zoo and Wildlife Research, Alfred-Kowalke Str. 17, D-10315 Berlin, Germany; 2 Institute of Virology, Helmholtz Zentrum München, Ingolstaedter Landstr. 1, D-85764 Neuherberg, Germany; 3 German Primate Center, Leibniz-Institute for Primate Research, Unit of Infection Models, D-37077 Göttingen, Germany; 4 III Medizinische Universitätsklinik, Medizinische Fakultät Mannheim der Universität Heidelberg, D-68305 Mannheim, Germany; 5 Institute of Toxicology, Helmholtz Zentrum München, Ingolstaedter Landstr. 1, D-85764 Neuherberg, Germany; 6 Molecular and Cell Physiology, Hannover Medical School, Carl-Neuberg-Str. 1, D-30625 Hannover, Germany

## Abstract

**Background:**

Prion diseases such as bovine spongiform encephalopathies (BSE) are transmissible neurodegenerative diseases which are presumably caused by an infectious conformational isoform of the cellular prion protein. Previous work has provided evidence that in murine prion disease the endogenous retrovirus (ERV) expression is altered in the brain. To determine if prion-induced changes in ERV expression are a general phenomenon we used a non-human primate model for prion disease.

**Results:**

Cynomolgus macaques (*Macaca fasicularis*) were infected intracerebrally with BSE-positive brain stem material from cattle and allowed to develop prion disease. Brain tissue from the *basis pontis *and *vermis cerebelli *of the six animals and the same regions from four healthy controls were subjected to ERV expression profiling using a retrovirus-specific microarray and quantitative real-time PCR. We could show that Class I gammaretroviruses HERV-E4-1, ERV-9, and MacERV-4 increase expression in BSE-infected macaques. In a second approach, we analysed ERV-K-(HML-2) RNA and protein expression in extracts from the same cynomolgus macaques. Here we found a significant downregulation of both, the macaque ERV-K-(HML-2) Gag protein and RNA in the frontal/parietal cortex of BSE-infected macaques.

**Conclusions:**

We provide evidence that dysregulation of ERVs in response to BSE-infection can be detected on both, the RNA and the protein level. To our knowledge, this is the first report on the differential expression of ERV-derived structural proteins in prion disorders. Our findings suggest that endogenous retroviruses may induce or exacerbate the pathological consequences of prion-associated neurodegeneration.

## Background

Prion diseases are fatal transmissible neurodegenerative diseases that include Creutzfeldt-Jakob Disease (CJD) in humans and bovine spongiform encephalopathy (BSE) in cattle [1-4]. A key observation in the development of prion diseases is the accumulation of the abnormal isoform (PrP^Sc^) of the host encoded prion protein (PrP^C^) in the brain of affected individuals [5,6]. As a result, neuronal loss and astrogliosis lead to the clinical symptoms associated with prion diseases such as ataxia and dementia. The mechanism by which PrP^C ^is converted to PrP^Sc ^is not well understood. As a result, several researchers have hypothesized that host-derived nucleic acids or other non-PrP molecules contribute to the propagation of prions [7-10]. In search of these unknown factors, multiple studies in mice and humans have examined host gene expression response to prion-infection revealing multiple biochemical pathways that are disturbed. These altered pathways may contribute to prion disease pathology [11-22] though most studies have yielded conflicting results among tissue types, methods, and cell lines used [23].

One group of sequences with altered expression as a consequence of prion infection in both, cell lines and tissue samples from humans and mice, are endogenous retroviruses (ERVs) [24-28]. ERVs are the remnants of germline infection by relatives of exogenous retroviruses such as murine leukemia virus (MLV) and mouse mammary tumor viruses (MMTV) [29]. Endogenization is a frequent event as the genomes of all mammals studied are composed of 8-11% retrovirus-like sequences. They are not only passive components of the genome or "Junk DNA", but can also play important functional roles. For example the ERV-derived proteins syncytin-1 and -2 are essential for trophoblast differentiation in placental development [30-33]. The long terminal repeats (LTRs), which control retroviral gene expression can also change host RNA levels [29]. Additionally, the ERV-expression pattern has been shown to be altered in a murine cell culture model for prion infection [25] and in the cerebrospinal fluid (CSF) of sporadic CJD patients [28]. The PrP and ERV expression in some immune-stimulated mouse tissues also appear to be linked [34]. Infections of mice with ecotropic neurovirulent retroviruses can also be associated with prion-like disorders. The MLV strains Cas-Br-E, Friend MLV Fr98 and the ts-1 mutant of MoMLV cause neuropathology that is similar to prion-induced spongiosis [35-37]. Interestingly, the prion gene is totally dispensable in the pathogenesis and tropism of Cas-Br-E [36]. This lead to the conclusion that retroviral-induced neurodegeneration may act in a common pathway but downstream of prion infection.

To date, the expression of endogenous retroviruses in human CJD has only been analysed in patients' CSF samples [28]. ERV profiling in brain tissue from human CJD has not been performed. A systematic analysis in a controlled experimental non-human primate model for prion disease provides the unique opportunity for examining the expression of ERVs in response to infectious prions in the primate brain. Cynomolgus macaques are an excellent model for human acquired prion disease. TSEs can be transmitted to *Macaca fascicularis *leading to disease that is comparable to humans, including preclinical incubation time, clinical symptoms and pathophysiology [38]. For ERV expression analysis, a microarray system for the detection of human endogenous retroviral expression has been successfully applied to genomic DNA and RNA derived from rhesus macaque tissues (*Macaca mulatta*) [39] or brain tissue from barbary macaques (*Macaca sylvanus*) [40].

The aim of our study was to assess the putative dysregulation of endogenous retroviruses on the transcriptional and protein level linked to prion pathology. For this reason we analyzed three defined brain regions, *vermis cerebelli, basis pontis *and the frontal/parietal cortex. The samples from the infected macaques were compared to samples from the same brain regions of non-infected control animals. Changes in gene expression were examined using a retrovirus-specific microarray [40] and quantitative real-time PCR (QPCR). Western blot analysis with an HERV-K-(HML-2) Gag specific antiserum was used to investigate changes at the protein level.

## Methods

### Tissues and RNA extraction

Cynomolgus macaques (*Macaca fascicularis*) were purchased from the Centre de Recherche en Primatologie, Mauritius and maintained in social groups of six monkeys housed in a microbiological containment level BSB3 facility. Ethics approval for the study was issued by the Lower Saxony Ministry for consumer protection and food safety (509.42502/08/07.98). Animal experimentation was performed in accordance with section 8 of the German Animal Protection Law in compliance with EC Directive 86/609. It should be mentioned that the diet and housing of the used experimental and the control animals were not identical. This may well have contributed to the inter-individual variations among the animals. Six cynomolgus macaques were intracerebrally inoculated with 50 mg bovine BSE-positive brain stem material derived from an EU-standard inoculum. Inoculation was performed by slow injection of 250 μl of a 20% BSE brain homogenate (w/v) diluted in sucrose into the right hemisphere through the dura mater into the caudo-putamen. The preclinical incubation time was on average 1,100 days. Animals were sacrificed, and the brains were dorso-ventrally sliced and snap-frozen on dry-ice plates. Brain material from age-and sex-matched non-infected cynomolgus macaques was obtained from Covance Laboratory Münster GmbH and processed in an equivalent manner. Three different brain regions (*vermis cerebelli, basis pontis*, frontal/parietal cortex) were macroscopically identified on the frozen brain slices, isolated via a bore device and ground to powder under liquid nitrogen (for extraction of RNA) or homogenized (for extraction of protein).

Total RNA (RNA > 200 bases) was isolated from each sample using a mirVANA kit (Ambion). To remove genomic DNA, all RNA samples were treated with 1 U/μg RQ1 RNase-free DNase I (Promega). Subsequently, 25 ng of each RNA preparation was tested by PCR with retroviral mixed oligo primers (MOP) as described previously [41] omitting the reverse transcription step. Only RNA preparations negative for amplification products were used for reverse transcription and MOP multiplex PCR. Samples tested positive were treated again with DNase I until the MOP multiplex control PCR were negative. This precaution was taken to ensure that all experiments measuring RNA levels were completely DNA-free.

### Reverse transcription, PCR and microarray analysis

Reverse transcription of RNA was performed using 1 μg total RNA with Superscript II (Roche Diagnostics) according to the manufacturer's protocol. Reverse transcriptions were followed by an RNase H digestion step (Promega). Syntheses of hybridization probes, labelling of MOP PCR products, preparation, hybridization and post-processing of retrovirus-specific DNA microarrays were performed as previously described [41,42]. The exception was that PCRs were performed with KAPA2G Robust DNA Polymerase (Peqlab Biotechnologie GmbH) which was found to improve the microarray results substantially in terms of reproducibility and signal intensity (data not shown).

Hybridized microarrays were scanned using an Affymetrix GMS 418 scanner (laser power setting, 100%; gain setting, 50%), and the resulting images (16-bit TIFF files) were subjected to densitometric analysis using ImaGene 4.0 software (BioDiscovery Inc.). To discriminate positive signals from background, a cut-off value of 1,200 relative fluorescence intensity units was used that corresponded to twofold overall background intensity value of the microarray. To account for the influence of RNA quality, HERV signals were normalized to the fluorescence intensity of the hypoxanthine-guanine phosphoribosyltransferase (HPRT) transcript as a housekeeping gene showing the most consistent transcript levels on the microarray. This was confirmed by densitometric analysis which demonstrated that there was no statistically significant difference in HPRT expression difference between infected and uninfected animals (data not shown). Thus, microarray-based relative HERV transcript levels were expressed as a ratio of HERV to HPRT. Microarray data were submitted to Gene Expression Omnibus (GEO: GSE29818).

### QPCR

Primers used for quantitative real time PCR (QPCR) were HERV-E: E4-1 (forward, 5'-GGTGTCACTACTCAATACAC-3'; reverse, 5'-GCAGCCTAGGTCTCTGG-3'), MacERV-4 (forward, 5'-ATGTAGCTGCCGCTATAGAG-3'; reverse, 5'-GCTATTTGTAGTCCGGCTGC-3') and ERV-K (HML-2) gag (forward, 5'- TATGATTGGGAGATTCTGGCA-3'; reverse, 5'-GCAGCCCTATTTCTTCGGAC-3'). The HERV-E, and MacERV-4 primers were previously shown to work on barbary macaques [40]. QPCR was performed with the Roche LightCycler480 System, using LC-480 SybrGreen PCR mix and a standard LightCycler protocol (Roche Diagnostics). Amplifications were performed using a 10 min denaturation step at 95°C, followed by 45 cycles of 10 sec at 95°C, 5 sec at 60°C, and 10 sec at 72°C. RNA-Polymerase II-transcripts (RPII) were analyzed as a standard, using published primers [43]. For quantification of ERV-K (HML-2) a 7500 Real Time PCR System (Applied Biosystem) was used. Reaction was performed after 10 min denaturation with 45 cycles between 95°C for 15 sec and 58°C for 45 sec using Power SybrGreen PCR Mastermix (Applied Biosystems) with 2.5 ng RNA equivalent amount and 400 nM of each primer per reaction. The relative expression ratio was calculated as described [44]. Melting curve analysis and gel electrophoresis of amplification products was performed to verify that artefact products or primer dimers were not responsible for the signals obtained (see Additional file 1: Figure S3).

### Western blot analysis

Frontal/parietal cortex samples were homogenized in sample buffer (62.5 mM Tris/HCl, pH 6.8, 62.5 mM Imidazol, pH 6.8, 10% SDS, 0.01% bromphenolblue, 12.5% glycerol, 2.5% beta-mercaptoethanol) at 20% (w/v) and stored at -80°C prior to use. Extracts were spun for 2 min at 7,000 × g and the supernatant was diluted 1:1 in LDS sample buffer (Invitrogen). 10% Glycerol (v/v) was added and samples were incubated for 5 min at 95°C and cooled on ice. For immunoblotting, 10 μl of supernatant was separated on a 12% Bis-Tris polyacrylamide gel in MOPS-SDS running buffer (NuPAGE, Invitrogen) together with a molecular weight marker (Spectra Multicolor Broad Range Protein Ladder, Fermentas) for 150 min at 30 mA. The gel was blotted onto a PVDF membrane (Immobilon-P transfer membrane 0.45 μm, Millipore) in transfer buffer (NuPAGE, Invitrogen) for 70 min at 135 mA. Prion infectivity of the blot was inactivated by incubation in 4 M Guanidinium-Thiocyanate (Roth) for 30 min at room temperature with slow agitation. The PVDF was washed in TBS and unspecific binding was blocked with 0.2% Casein (I-Block, Applied Biosystems) for 30 min at room temperature. As primary antibody, a polyclonal antiserum (K2548) that was raised against full length HERV-K-(HML-2) Gag [45] was used at a dilution of 10^-2^. This antiserum was generated as described for serum 6897 [45]. A pre-immune serum derived from the same animal was used as a negative control. As a loading control, a β-actin antibody (mAbcam 8224) was used at a dilution of 10^-3^. Antibody incubation was performed overnight at 4°C in TBS/0.02% Casein. Detection was carried out using anti-rabbit IgG-AP (A9919, Sigma-Aldrich) at a dilution of 10^-5 ^in TBS/0.02% Casein for 1 h at room temperature with gentle agitation. Bands were visualized with NBT/BCIP substrate (Moss Inc.) for 10 min. The reaction was stopped by addition of ddH_2_O and scanned. Densitometric analysis was performed with the program Image J 1.37 v.

### Statistics

Data analysis was performed using GraphPad Prism 5.0. Significance was calculated using unpaired student t-test. The corresponding results are indicated in the figures (***, p < 0.001; **, p < 0.01; *, p < 0.05).

## Results

### ERV expression profiling in two distinct brain regions of cynomolgus macaques

We investigated whether the expression of endogenous retroviruses was influenced by the infection with prions. RNA was prepared from brain tissue of BSE-infected and non-infected macaques at an advanced stage of prion disease and subjected to reverse transcription with random hexamer primers. Prior to cDNA syntheses, the DNA-free status of each RNA sample was confirmed to prevent interference from DNA copies of ERVs with the microarray probes. It should be noted that the microarray detects HERV groups and not individual HERVs. Thus, signals arising from a HERV group do not necessarily identify specific elements responsible for the signal.

**Figure 1:**
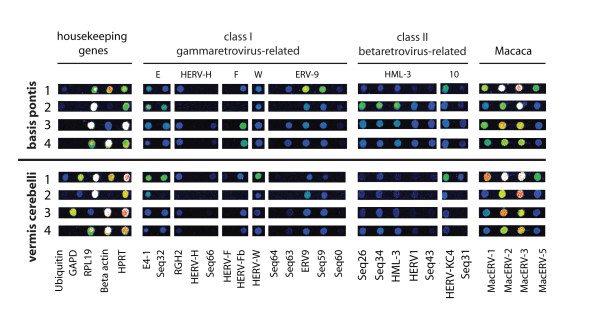
**Core ERV expression profile of macaque brain regions *basis pontis *and *vermis cerebelli***. Fluorescence signal intensities of ERV-specific microarray spots from four (1-4) non-infected individuals are shown as false colour images. Individual ERV names have been described earlier [42].

Initially we investigated the general expression, or core profile, of ERVs derived from four non-infected cynomolgus macaques in two distinct brain regions, the *basis pontis *and the *vermis cerebelli *(Figure 1, for a full profile also see Additional file 1). The vast majority of the human derived oligonucleotides on the microarray cross-hybridized with macaque ERVs as has been observed for Old World Monkeys in general [39]. ERV profiling revealed high expression of class I gammaretroviruses HERV-E (E4-1, Seq32), HERV-F (HERV-Fb), HERV-W, ERV9 (ERV9, Seq59), class II betaretroviruses HERV-K-(HML-3: Seq26, Seq34, HML-3), HERV-K-(HML-10): (HERV-KC4) and macaque specific ERVs (MacERV-1, -2, -3, and -5). Among the animals we detected inter-individual differences in the respective expression pattern. Such differences have also been reported for human studies [46], likely as a result from the non-inbred genetic background of the tested individuals. However, the respective profiles of *basis pontis *and *vermis cerebelli *regions were comparable among the macaques.

Comparison of the derived ERV-expression profile in macaques to that of healthy human brain samples [46] revealed a partial overlap of the expressed ERVs including HERV-E4-1, ERV9, Seq59 and HERV-KC4. In contrast, HML-3 elements Seq26, Seq34 and Seq43, that were not or only barely detectable in human prefrontal cortex [46] were highly expressed in *basis pontis *of macaques. Our results are consistent with previous studies on both DNA and RNA from other non-human primate species that showed both generally conserved presence and expression patterns for several ERVs and additional species specific patterns [39,40].

### Differential expression of ERVs in BSE-infected macaques

**Figure 2:**
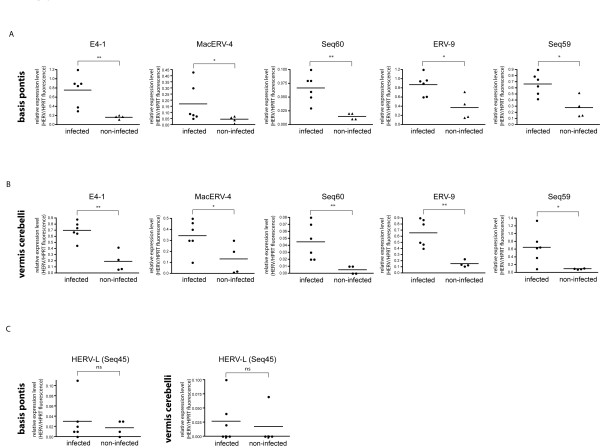
**Microarray analysis of ERV-expression in BSE-infected compared to non-infected macaques**. Fluorescence signal intensities from six BSE-infected macaques were compared to four non-infected controls. Relative expression was calculated as the ratio to the expression of the housekeeping gene HPRT. Results for ERV-E4-1, MacERV-4 and ERV-9 group members Seq60, ERV-9 and Seq59 are shown for *basis pontis ***(A)** and *vermis cerebelli ***(B)**. Statistical significance was analysed using the unpaired students t-test (** p < 0.01; * p < 0.05; ns, not significant). **(C) **shows non-regulated HERV-L (Seq45) in both brain regions.

Next we used the retrovirus-specific microarray to compare the ERV-expression patterns of BSE-infected with that of non-infected macaques. Quantitative comparison in two different brain regions revealed statistically significant differences between BSE-infected and non-infected macaques (Figure 2). In the infected animals pathogenic prion protein (PrP^Sc^) was detected in the *vermis cerebelli *and *basis pontis *as confirmed by Western blot analysis (data not shown). A detailed description of the distribution of macaque-adapted BSE will be reported elsewhere. Increased expression in BSE-affected macaques was detected for class I elements ERV-E4-1, the macaque specific MacERV-4 and the ERV-9 elements Seq60, ERV9, Seq59. It should be noted that, although statistically significant, upregulation of MacERV-4 seemed to be mainly driven by outliers, as compared to the robust increase of expression in E4-1 and ERV-9 indicating that for some affected ERVs, there is interidividual variability in response to prions. However, upregulation of these endogenous retroviruses was detected in both, *basis pontis *and *vermis cerebellis*.

Class II element ERV-K-(HML-3) Seq43 was statistically significant by microarray but also driven by outliers (Additional file 1: Figure S2). None of the Class III HERV-L related elements showed a consistent change in expression. This was not surprising as they are related to foamy viruses which do not cause any known pathological effects as exogenous viruses and are widespread in the genome of mammals indicating they have been endogenized for tens of millions of years. No other changes in ERV expression reached statistical significance for either brain region.

### Validation of differentially expressed ERVs in BSE-infected macaques by QPCR

**Figure 3:**
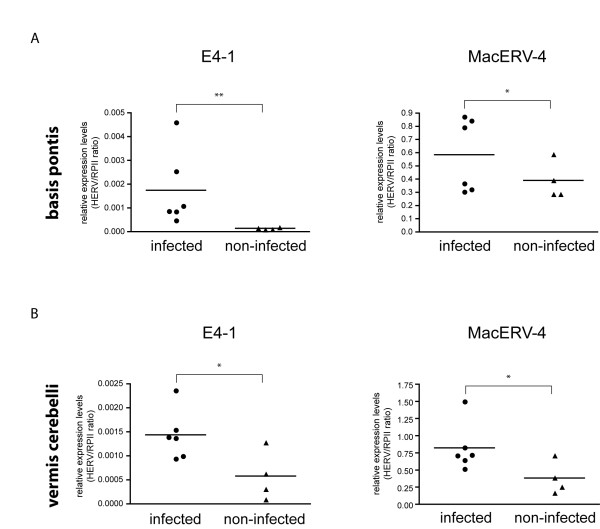
**Relative quantification of ERV-expression in BSE-infected versus non-infected macaques using QPCR**. Quantitative expression of ERV-E4-1 and MacERV-4 were assessed by QPCR. Expression was normalized to the housekeeping gene RPII. Points represent the mean of two experiments in duplicates is shown. Statistical significance was determined by unpaired students t-test (*** p < 0.001; ** p < 0.01; * p < 0.05).

The retrovirus-specific microarray is a powerful tool for the comprehensive expression analysis and comparison of multiple ERVs in one single experiment. However, the approach cannot differentiate between individual group members of the tested ERVs. Consequently the specificity of hybridization data can be increased by the use of assays designed for the quantification of defined transcripts in a given sample. To confirm the microarray results for HERV-E4-1 and MacERV-4 specific PCR primers were designed. To confirm the specificity of the chosen oligonucleotides the generated PCR products were checked for specific products of the correct size after amplification from cynomolgus macaque-derived cDNA (data not shown) prior to performing the quantitative assays. The same was done for the housekeeping gene chosen, RNA polymerase II (RPII). The primers for HERV-E4-1 have been successfully applied to barbary macaques [40]. Using QPCR we could confirm a significantly elevated HERV-E4-1 expression in the BSE-infected animals as compared to the non-infected controls in the *basis pontis *(Figure 3A) as well as in the *vermis cerebelli *(Figure 3B). In addition HERV-K-(HML-3) and the macaque-specific ERV MacERV-4 showed a statistically significant increase in expression upon BSE-infection in both tested brain regions. Since the control animals were not mock infected we cannot rule out that expression of endogenous retroviruses was influenced by the inoculation procedure itself. However, the regions used for our study were spatially separated from the site of inoculation. In addition, our expression analysis was performed approximately 3 years post inoculation which virtually excludes persistent effects triggered by injection of tissue homogenate. In summary, we have identified and validated three ERVs that exhibit an increased expression upon BSE-infection of cynomolgus macaques using two independent techniques, microarray and QPCR. We could show that this differential expression exhibited comparable levels in both of the investigated regions, *basis pontis *and *vermis cerebelli*.

### Comparison of ERV-K-(HML-2) Gag protein levels in the frontal/parietal cortex from BSE-infected and non-infected macaques

**Figure 4:**
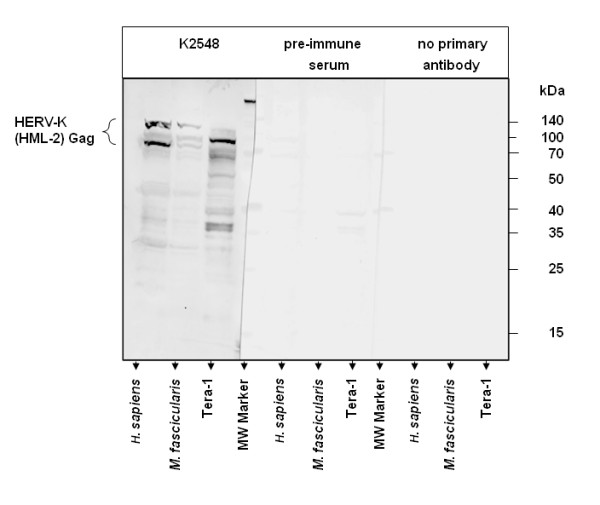
**Specific detection of ERV-K-(HML-2) Gag in brain samples derived from macaques and humans**. Extracts from the frontal/parietal cortex region of *M. fascicularis *and *H. sapiens*, respectively, and lysates from Tera-1 cells (kindly provided by Dr. J. Mayer) were separated by PAGE and blotted to PVDF membrane. Detection was performed with polyclonal antibody K2548 (left panel), pre-immune serum (middle) or by omission of the primary antibody (right panel), respectively. Predominant HERV-K-(HML-2) Gag immunoreactivity was detected at 70, 80 and 105 kDa, but was absent in the pre-immune and secondary antibody controls. Molecular weight marker bands are indicated at the right.

Only a few antibodies against ERVs are commercially available. Even fewer have been tested against non-human primates. Unfortunately, to our knowledge, antibodies directed against polypeptides encoded by the BSE-induced ERVs HERV-E4-1, HERV-K-(HML-3), ERV-9 and MacERV-4 do not exist. We tested a panel of ERV-antibodies (kindly provided by J. Mayer) against *Homo sapiens *ERV-K-(HML-2) Gag and Env for cross-reactivity with macaque ERV-K-(HML-2) proteins in cortical samples from *Macaca fascicularis*. Most of the tested antibodies showed low specificity (K1182, polyclonal) or no signal (monoclonal antibodies 6E2-1-4, 3H11 and HERM-1831-5) (data not shown). However, polyclonal antibody K2548 that has been raised against HERV-K-(HML-2) Gag [45] exhibited specific bands (Figure 4). Proteins at 70, 80 and 105 kDa, respectively, showed an identical migration pattern in simian brain as compared to a human brain sample. Furthermore, the band at 80 kDa coincides with the 80 kDa full-length Gag protein that is secreted from teratocarcinoma cells (Tera-1) cells [47]. Both, incubation with secondary antibody alone and the respective pre-immune serum did not produce any signal in the respective molecular weight range, confirming that the K2548 antiserum specifically detects macaque ERV-K-(HML-2) Gag. Using this panel of positive and negative controls we concluded that antiserum K2548 could specifically detect macaque ERV-K-(HML-2) Gag. It should be mentioned that the probes and antisera used in our study were not developed for the use in cynomolgus macaques. However, macaques and humans are very closely related species on the DNA and on the protein level. In addition, we have demonstrated that the used antiserum detects the same proteins in human and *Macaca fascicularis *brain samples. This suggests that the cross-reactivity is sufficient to support our conclusions.

**Figure 5:**
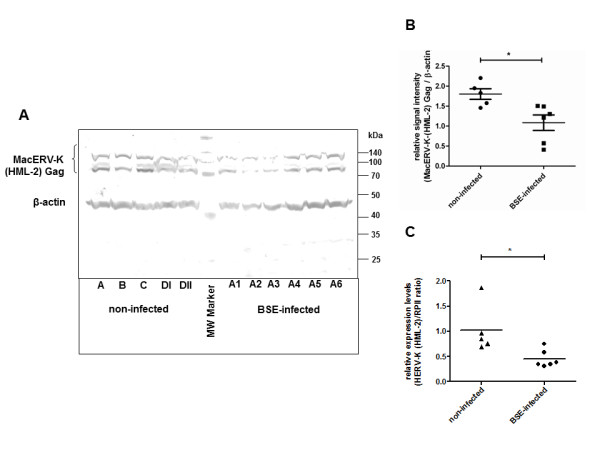
**Reduced expression of macaque ERV-K-(HML-2) Gag in brains of BSE-infected macaques**. **(A)**. Extracts derived from the frontal/parietal cortex of BSE-infected (right) or non-infected (left) of *Macaca fascicularis *were subjected to PAGE, blotted to PVDF membrane and incubated with K2548. Immunoreactivity was visualized with NBT/BCIP. For loading control the blot was co-incubated with a monoclonal beta-actin antibody. **(B)**. Densitometric analysis of A. NBT/BCIP-intensities of HERV-K-(HML-2) Gag were normalized to beta-actin intensities. Statistical analysis was performed using the unpaired students t-test (* p < 0.05). **(C) **Quantitative expression of ERV-K-(HML-2) assessed by QPCR. Expression was normalized to the housekeeping gene RPII. Points represent the mean of two experiments in duplicates. Statistical significance was determined by unpaired t-test (* p < 0.05).

We next addressed the question whether macaque ERV-K-(HML-2) Gag protein was differentially expressed in BSE-infected compared to non-infected macaques. Western blot analysis revealed a considerably lower abundance of macaque ERV-K-(HML-2) Gag in the prion-infected animals (Figure 5A). To quantify the difference in the protein levels we performed a densitometric analysis. Normalization to the co-detected beta-actin revealed that macaque ERV-K-(HML-2) Gag protein expression was significantly decreased in BSE-infected macaques as compared to non-infected individuals (Figure 5B). This reduction was confirmed in three independent Western blot experiments and by densitometric analysis of total protein staining using Ponceau S (data not shown).

To analyse whether the downregulation was mirrored at the RNA-level we performed a macaque ERV-K-(HML-2) *gag *specific QPCR on RNA samples derived from the frontal/parietal cortex. A significant downregulation of ERV-K-(HML-2) RNA expression was observed in infected animals, which is consistent with the protein expression data for this brain region (Figure 5C). To our knowledge this is the first report of the regulation of an endogenous virus protein coinciding with prion disease in cynomolgus macaques as a model for Creutzfeldt-Jacob disease.

## Discussion

Although regarded as a part of the "Junk DNA", ERVs have been shown to play unique functional roles in cellular biology and are implicated in a wide range of diseases like cancer, autoimmune disease, and neurodegeneration [48-51]. We first compared the expression profiles of our old world monkeys (*Macaca fascicularis*) to existing human profiles [41,46,52]. Beside a broad number of similarities, we identified differences in the expression profiles between humans and macaques when comparing Class II betaretroviruses. The HERV-K-(HML-2, -6, and -9) that are strongly expressed in human brain are at the detection limit in macaque brain.

This result is consistent with prior studies of Old World Monkey ERV expression in brain compared to human using a comparable microarray and QPCR [40]. In addition, HERV-K-(HML-3) is more strongly expressed in the *basis pontis *as compared to the *vermis cerebelli *of cynomolgus macaque brain. This shows that regional specific differences in ERV expression for class II beta-retroviruses exist in macaques arguing for a functional relevance of these transcripts. We cannot judge whether this finding depicts a general phenomenon. However, the brain region specific expression of ERVs may provide additional insights into molecular mechanisms regulated by ERVs.

Using our non-human primate model we next examined the regulation of endogenous retrovirus expression upon BSE-infection. To date, prion-infected macaques have not been used for analysis of ERV-expression. Differential expression of ERVs upon prion disease have been previously reported in other species, including humans, though brain material has not been examined [28]. Here we provide evidence that ERVs are differentially expressed in an experimentally controlled macaque model of BSE-infection which is highly relevant for human CJD. We have identified the upregulation of ERVs E4-1, MacERV-4, and ERV-9 by ERV-specific microarray. Interestingly, ERV-9 has also been shown to be upregulated in cerebral spinal fluid of sCJD patients [28]. Thus, the macaque-adapted BSE and human sCJD profiles show common ERV-regulation patterns in response to prion infection irrespective of the analysed sample types (brain or cerebrospinal fluid).

We could not judge whether the regulation of ERVs is cause or consequence of prion infection. Although many *in vivo *studies have been performed, the mechanism connecting prion deposition and neuronal loss has not been determined in detail. The spatial distribution of PrP^Sc^-aggregates in different brain regions is not always coincident with neuronal decay in these areas. Thus it appears that neurodegeneration itself could be promoted by biological pathways that are more complex than protein aggregation, possibly by analogous mechanisms as described for neurotropic retroviruses. Indeed, it is known that some retroviruses can induce neurodegeneration in the absence of infectious prions. Different classes of leukemia retroviruses, including CasBrE, 10A1-MuLV, ts1MoMuLV-TB and MoAmphoV induce fatal spongiform encephalomyelopathy [35-37]. Symptoms include tremor, wasting and paraparesis that is caused by neuronal loss, spongiform lesions and astrogliosis, which is intriguingly similar to prion disease pathology [53]. All four retroviruses are classified as gammaretroviruses, the same subgroup as the here described BSE-upregulated MacERV-4, ERV-9 and HERV-E. Thus, changes in ERV expression may induce or exacerbate the pathological consequences of neurodegeneration.

Upregulation of endogenous retroviruses can also potentially induce neurodegeneration. It has been shown that the regulatory LTR-region of HERV-E regulates the expression of the Opitz-syndrome associated *mid1 *gene [54] that is essential for *vermis cerebelli *development [55]. It has been proposed that overexpression of *mid1 *leads to disturbance in microtubule-homeostasis, comparable to the tau-aggregation induced neuronal loss in Alzheimer's disease [56,57]. Thus upregulation of HERV-E in BSE-infected macaques may also contribute to neuronal decay similar to the above mechanism. This suggests that induction of retroviral elements may be a consequence of prion infection, and a downstream mechanism of neurodegeneration [36]. This, however, does not imply that endogenous retroviruses can cause prion disease. The appearance of viral particles in prion-infected cells [58,59] has been discussed controversially. Interestingly, recent work has identified PrP^C ^as an integral component of the Human Immunodeficiency Virus-1 (HIV-1) [60]. After cells are infected with HIV-1, the virus replicates, assembles and buds preferentially from the lipid rafts [61] presumably including the GPI-anchored PrP^C ^into the surface of the HIV-1 particles. In addition, HIV-1 Gag and Env colocalize with PrP^C ^in infected T-cell lines [62]. Furthermore, expression of HIV-1 *gag *increases the susceptibility to and sustains the prion infection in cell culture [26,63]. Thus spreading of prion infection from one cell to another may not be restricted to exosomal vesicle transfer [64], but may also be triggered by Gag of endogenous origin, such as MacERV-K-(HML-2) Gag. In line with this, murine N2a cells express, produce, and release MuLV (murine-leukaemia virus) particles of endogenous origin, called NeRV (neuroblastoma endogenous retrovirus) [65]. Infection of N2a cells with prions leads to the excretion of exosomes that harbor PrP^C ^or infectious PrP^Sc^. Intriguingly, antibodies against PrP^C ^could label both, exosomes and infectious virions [66]. This suggests that intercellular trafficking of prions could at least partially be mediated by hitchhiking on endogenous retroviral particles. This is in line with earlier observations that PrP can interact with retroviral RNA [67-69] eventually resulting in the formation of active nucleoprotein structures [70] that include Gag. Using specific antibodies we could show that macERV-K-(HML-2) Gag protein is expressed in the brains of cynomolgus macaques. To our knowledge this is the first time that a structural element of an endogenous retrovirus has been detected in the primate central nervous system. The functional role of the protein in neuronal physiology is unknown. Interestingly, among the many endogenous retroviral subfamilies, HERV-K-(HML-2) can produce viral particles [71-74]. It remains unclear whether macERV (HML-2) Gag can be excreted by neurons. However, active release of HERV-K-(HML-2) Gag containing retrovirus-like particles have been described in Tera-1 [47] cells and were also found in blood plasma of patients [75,76]. Taken together, the detection of macERV-K-(HML-2) protein in the frontal/parietal cortex indicates that it may have a physiological role in the brain. Furthermore, finding that HERV-K-(HML-2) Gag protein and RNA is downregulated in BSE-infected macaques suggests that this role may be connected to neuronal survival. Further studies will be necessary to determine the mechanism and function of HERV-K HML-2 Gag downregulation.

## Conclusions

We could show that prion disease in a non-human primate model is associated with the alteration of ERV-expression at the transcriptional and the protein level. Upregulated expression of ERVs in macaques demonstrated partial overlap to those found in CSF from human CJD patients, underlining that our model is especially suitable to mimic the pathology of human TSEs. This is consistent with the hypothesis that endogenous retroviruses may provide a missing link between protein aggregation and neuronal loss in prion diseases. It is reasonable to speculate that the putatively secreted HERV-K (HML-2) Gag may contribute to the survival of neuronal cells.

Based on our findings, further research using specialized models may help to elucidate the association between prions and endogenous retroviruses and its putative role in neurodegeneration.

## Competing interests

The authors declare that they have no competing interests.

## Authors' contributions

ADG and MOZ designed the study. MV and WS established the microarray and carried out the experiments. MV carried out the QPCR experiments. ACS carried out the Western Blot analysis. JM carried out the RNA sample preparations from simian tissues. MV and ACS were responsible for the data analysis. ADG, JM and MOZ wrote the manuscript with contributions from all authors. All authors read and approved the final manuscript.

## Supplementary Material

Additional file 1 **Figures S1, S2, and S3**. Click here for file
