# Supplementary material for: Bovine spongiform encephalopathy infection alters endogenous retrovirus expression in distinct brain regions of cynomolgus macaques (Macaca fascicularis)
Source: Mol Neurodegener. 2011 Jun 23;6:44. doi: 10.1186/1750-1326-6-44 (PMC3152937; doi:10.1186/1750-1326-6-44)
Supplement: Additional file 1 — Figures S1, S2, and S3. [file 1750-1326-6-44-S1.PDF]

Additional file 1: Figures S1, S2, and S3

Bovine spongiform encephalopathy infection alters endogenous retrovirus expression in distinct brain regions of cynomolgus macaques (*Macaca fascicularis*)

Alex D. Greenwood[1,†], Michelle Vincendeau[2,†,‡], Ann-Christin Schmädicke[3], Judith Montag[3,#], Wolfgang Seifarth[4] and Dirk Motzkus[3,*]

[1)] Leibniz-Institute for Zoo and Wildlife Research & Faculty of Veterinary Medicine, Freie Universität Berlin, Alfred-Kowalke Str. 17, D-10315 Berlin, Germany.

[2)] Institute of Virology, Helmholtz Zentrum München, Ingolstaedter Landstr. 1, D-85764 Neuherberg, Germany.

[3)] German Primate Center, Leibniz-Institute for Primate Research, Unit of Infection Models, D-37077 Göttingen, Germany.

[4)] III Medizinische Universitätsklinik, Medizinische Fakultät Mannheim der Universität Heidelberg, D-68305 Mannheim, Germany.

[‡] Current address: Institute of Toxicology, Helmholtz Zentrum München, Ingolstaedter Landstr. 1, D-85764 Neuherberg, Germany

[#] Current address: Molecular and Cell Physiology, Hannover Medical School, Carl-Neuberg-Str. 1, D-30625 Hannover.

[†] Both authors contributed equally to this work

* To whom correspondence should be addressed at the German Primate Center, Leibniz-Institute for Primate Research, Unit of Infection Models, D-37077 Göttingen, Germany, Tel.: +49-551-3851-295, Fax: +49-551-3851-184, dmotzkus@dpz.eu

Email addresses:

ADG: greenwood@izw-berlin.de
MV: michelle.vincendeau@helmholtz-muenchen.de
ACS: schmaedicke@dpz.eu
JM: Montag.Judith@mh-hannover.de
WS: Wolfgang.Seifarth@medma.uni-heidelberg.de
MOZ: dmotzkus@dpz.eu

FIGURE S1

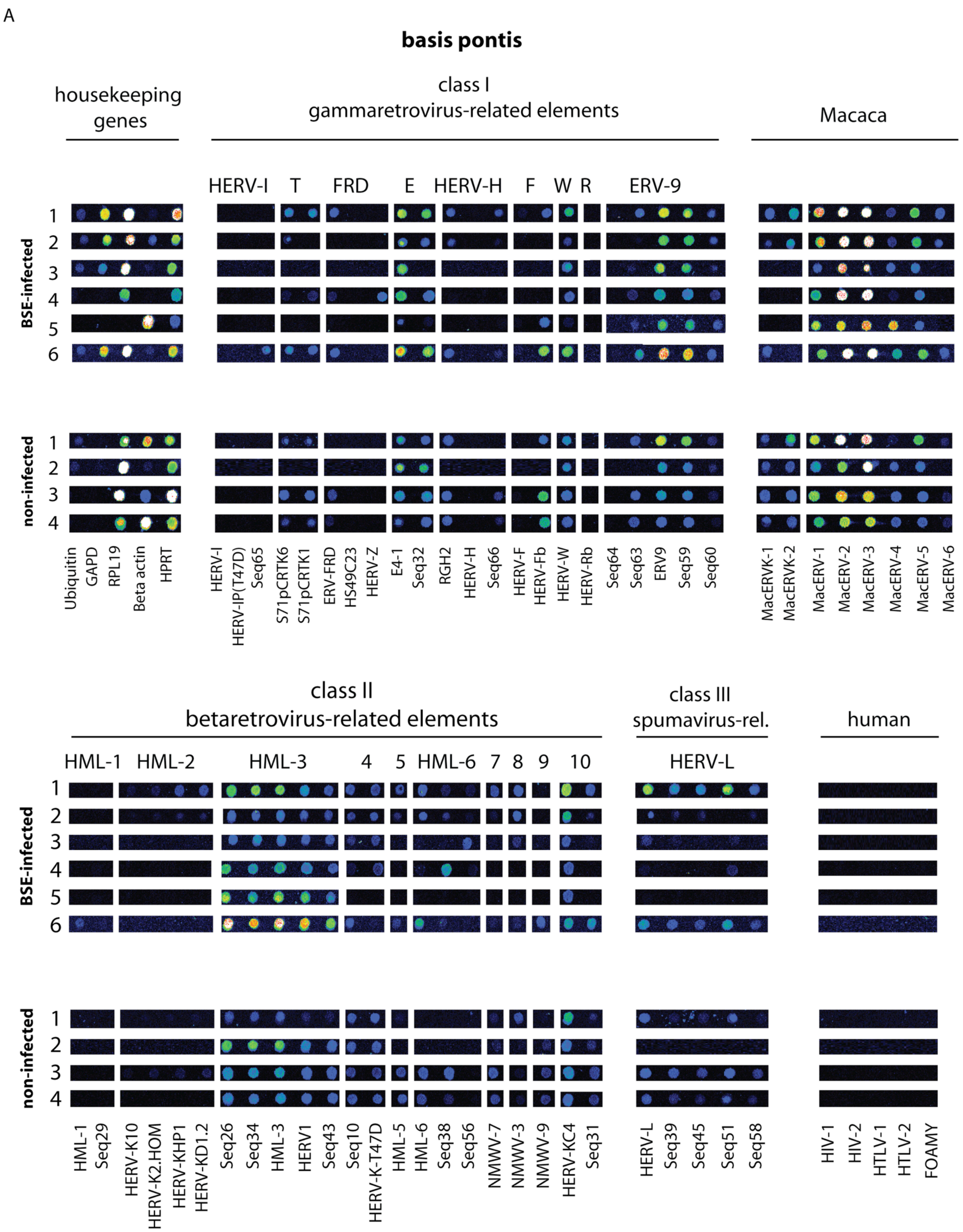
A
basis pontis
housekeeping genes
class I
gammaretrovirus-related elements
Macaca
HERV-I
T
FRD
E
HERV-H
F
W
R
ERV-9
BSE-infected
non-infected
1
2
3
4
5
6
Ubiquitin
GAPD
RPL19
Beta actin
HPRT
HERV-I
HERV-IP(T47D)
Seq65
S71pCRTK6
S71pCRTK1
ERV-FRD
HS49C23
HERV-Z
E4-1
Seq32
RGH2
HERV-H
Seq66
HERV-F
HERV-Fb
HERV-W
HERV-Rb
Seq64
Seq63
ERV9
Seq59
Seq60
MacERVK-1
MacERVK-2
MacERV-1
MacERV-2
MacERV-3
MacERV-4
MacERV-5
MacERV-6
class II
betaretrovirus-related elements
class III
spumavirus-rel.
human
HML-1
HML-2
HML-3
4
5
HML-6
7
8
9
10
HERV-L
HML-1
Seq29
HERV-K10
HERV-K2.HOM
HERV-KHP1
HERV-KD1.2
Seq26
Seq34
HML-3
HERV1
Seq43
Seq10
HERV-K-T47D
HML-5
HML-6
Seq38
Seq56
NMWV-7
NMWV-3
NMWV-9
HERV-KC4
Seq31
HERV-L
Seq39
Seq45
Seq51
Seq58
HIV-1
HIV-2
HTLV-1
HTLV-2
FOAMY

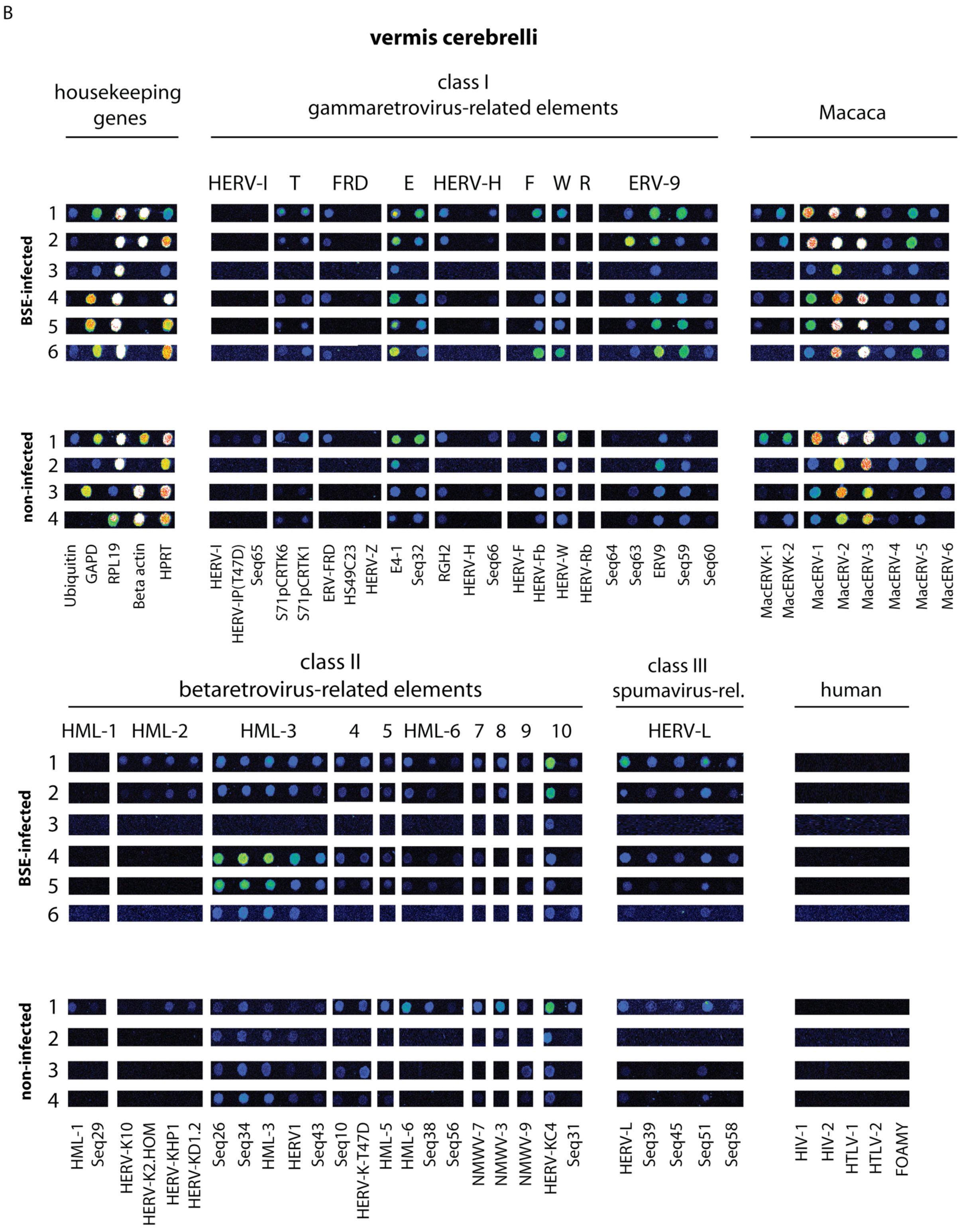
B
vermis cerebrelli
housekeeping genes
class I gammaretrovirus-related elements
Macaca
HERV-I
T
FRD
E
HERV-H
F
W
R
ERV-9
BSE-infected
non-infected
1
2
3
4
5
6
Ubiquitin
GAPD
RPL19
Beta actin
HPRT
HERV-I
HERV-IP(T47D)
Seq65
S71pCRTK6
S71pCRTK1
ERV-FRD
HS49C23
HERV-Z
E4-1
Seq32
RGH2
HERV-H
Seq66
HERV-F
HERV-Fb
HERV-W
HERV-Rb
Seq64
Seq63
ERV9
Seq59
Seq60
MacERVK-1
MacERVK-2
MacERV-1
MacERV-2
MacERV-3
MacERV-4
MacERV-5
MacERV-6
class II betaretrovirus-related elements
class III spumavirus-rel.
human
HML-1
HML-2
HML-3
4
5
HML-6
7
8
9
10
HERV-L
HML-1
Seq29
HERV-K10
HERV-K2.HOM
HERV-KHP1
HERV-KD1.2
Seq26
Seq34
HML-3
HERV1
Seq43
Seq10
HERV-K-T47D
HML-5
HML-6
Seq38
Seq56
NMWV-7
NMWV-3
NMWV-9
HERV-KC4
Seq31
HERV-L
Seq39
Seq45
Seq51
Seq58
HIV-1
HIV-2
HTLV-1
HTLV-2
FOAMY

FIGURE S1. Full profile of ERV expression. Alignment of false-color chip data sets corresponding to HERV classes I, II, and III transcriptional activity observed for all animals tested in this study and for each HERV analyzed on the microarray described in reference (24). Housekeeping genes served as an internal control for mRNA integrity. For data consistency, two replicas of the capture probe set were present on each DNA chip. Assays were carried out three times. The data for the *basis pontis* is shown in A and the *vermis cerebelli* in B.

HERV-K-(HML-3)

The Class II HERV, HERV-K-(HML-3) seq43 was determined to be statistically significant by densitometric analysis of the microarray (FIGURE S2 A). Given that the HML-3 group is one of the few strongly expressed groups in macaque brain and the profiles appear visually similar among the HERVs, we postulated that the microarray may be underestimating the expression differences between infected and uninfected animals for this complex ERV group. Using primers for HML-3 seq26 that have been shown previously to work for QPCR in barbary macaques (forward, 5'-CTGCAGCCTGCTAAGCG-3'; reverse, 5'–CACTGTGAAAATTTTTACGAG-3') (40), the QPCR results demonstrated statistical significance for seq26 (FIGURE S2 B). This suggests that additional HML3 elements are upregulated by BSE infection. However, in both the array and QPCR experiments, the results were only marginally significant suggesting that a much larger cohort of animals would be necessary to determine how differentially regulated HML-3 sequences are in general in macaques. This contrasts with the clearer statistical results of E4-1, MacERV4 and the ERV9 elements.

FIGURE S2

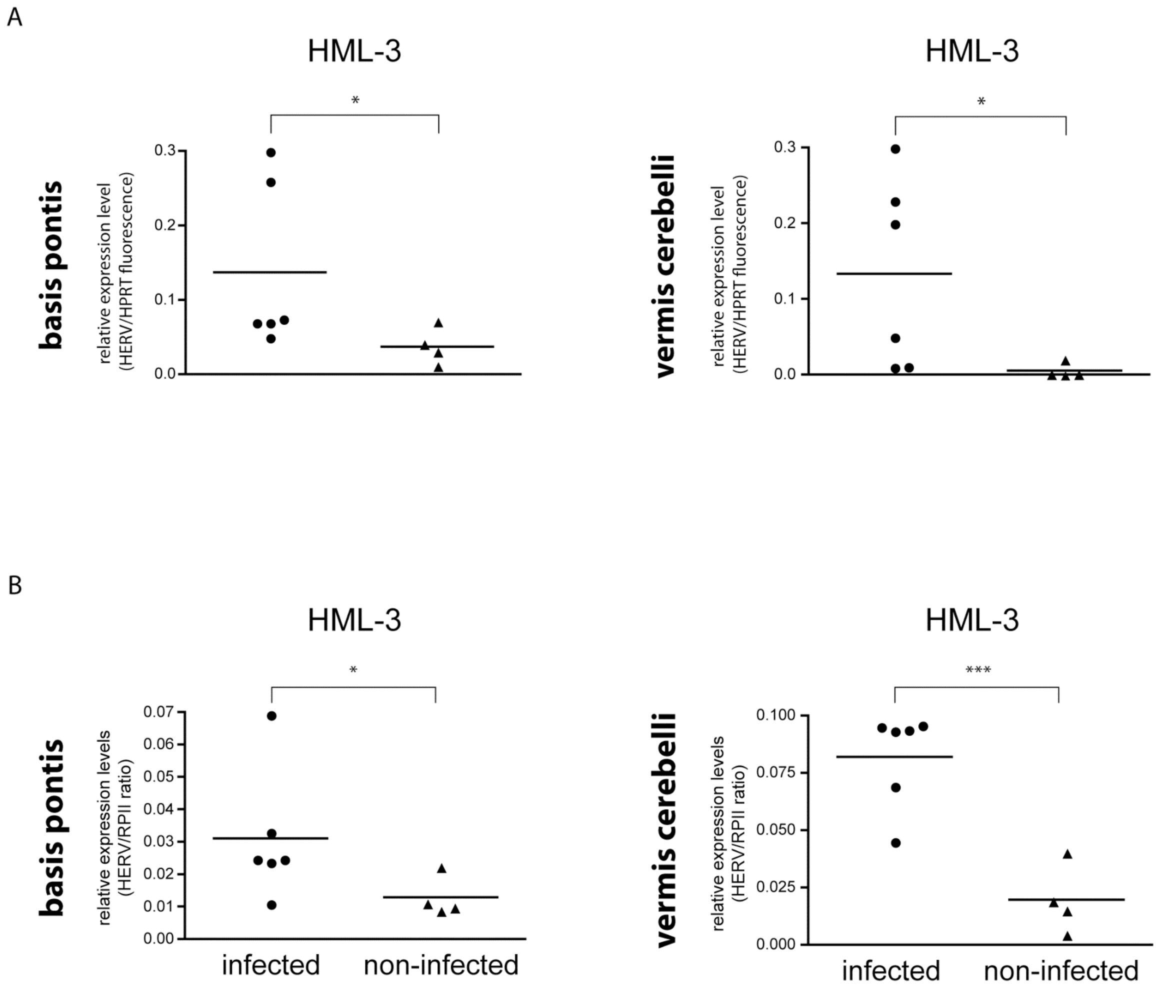

FIGURE S2. The microarray densitometric analysis of HERV-K-(HML-3) seq43 in the *basis pontis* and *vermis cerebelli* is shown in panel A. The QPCR results for the same regions for HERV-K-(HML-3) seq26 are shown in panel B.

FIGURE S3

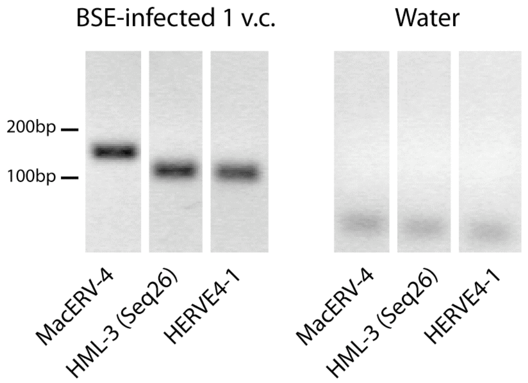

FIGURE S3. The Figure shows the gel profile of QPCR amplification products for MacERV-4, HML-3 and E4-1 in the BSE-infected animal 1 (*vermis cerebelli*). The expected sizes of 160bp (MacERV-4), 121bp (HML-3) and 119bp (E4-1) could be amplified. In the water control no product could be detected.
